# Correction: Waste or Resource? Investigating the interplay of structural support and individual attitude on council-based waste managers household food waste intervention

**DOI:** 10.1371/journal.pone.0335570

**Published:** 2025-10-28

**Authors:** The PLOS One Staff

## Notice of Republication

This article was republished on October 14, 2025 to correct an error in the title that was introduced during the production process. The publisher apologizes for the errors. Please download this article again to view the correct version. The originally published, uncorrected article and the republished, corrected articles are provided here for reference.

## Supporting information

S1 File Originally published, uncorrected article. (PDF)

S2 File Republished, corrected article. (PDF)
