# Supplementary material for: Correction: Waste or Resource? Investigating the interplay of structural support and individual attitude on council-based waste managers household food waste intervention
Source: PLoS One. 2025 Oct 28;20(10):e0335570. doi: 10.1371/journal.pone.0335570 (PMC12561898; doi:10.1371/journal.pone.0335570)
Supplement: S1 File — (PDF) [file pone.0335570.s001.pdf]

PLOS ONE

RESEARCH ARTICLE

Waste or resource? Investigating the interplay of structural d waste managers' household food waste interventions

Esther Landells[1,2]*, **Gamithri G. Karunasena**[2,3], **Samuel Oakden**[4], **Anjum Naweed**[1]

1 Central Queensland University, Appleton Institute for Behavioural Science, Wayville, South Australia, Australia, **2** End Food Waste Cooperative Research Centre, Adelaide, Australia, **3** Central Queensland University, School of Business and Law, Sydney, Australia, **4** Stop Food Waste Australia, Adelaide, Australia

* esther.landells@cqumail.com

Check for updates

OPEN ACCESS

Citation: Landells E, Karunasena GG, Oakden S, Naweed A (2024) Waste or resource? Investigating the interplay of structural d waste managers' household food waste interventions. PLoS ONE 19(7): e0303391. https://doi.org/10.1371/journal.pone.0303391

Editor: Sadia Ilyas, Hanyang University - Seoul Campus: Hanyang University, REPUBLIC OF KOREA

Received: June 13, 2023

Accepted: April 24, 2024

Published: July 26, 2024

Copyright: © 2024 Landells et al. This is an open access article distributed under the terms of the Creative Commons Attribution License, which permits unrestricted use, distribution, and reproduction in any medium, provided the original author and source are credited.

Data Availability Statement: All relevant data are within the manuscript.

Funding: The author received no specific funding for this work.

Competing interests: The authors have declared that no competing interests exist.

Abstract

Globally, food waste is a significant environmental, economic and social issue. Food waste in landfill creates the potent greenhouse gas, methane, contributing to climate change, with its management predominantly falling to local governments. Despite efforts by many countries, and extensive infrastructure and market development funding, food waste continues to be landfilled, with apparently similar councils taking diverse approaches. Using a mixed methods study design, data was firstly collected from a National online survey of Australian council-based waste management staff (n = 183), with descriptive and factor analysis of the survey data revealing a strong sense of structural empowerment (PCA.75 to.90) and a preference for sourcing information from networks (48%). These results informed a series of semi-structured interviews (n = 43) which, after thematic analysis, provided rich insights into the attitudinal and situational interpretations council-based waste managers bring to decisions around household food waste management. Framed by four pillars of Structural Empowerment, the findings suggest that waste manager's attitude is equally as important as support, resources, and knowledge and that, despite mandates and targets, individual motivational factors and organisational paradigms determined decision-making. Identified barriers included perceived capacity constraints, inadequate focus on actionable interventions, and fragmented, uninspiring, planning. This article provides important insights around 1) leveraging networks for knowledge dissemination, 2) fostering capacity-building initiatives, and 3) advocating for sustained engagement with food waste diversion within councils. This underscores the need for additional research into evolving council typologies and effectively engaging key stakeholders to achieve food waste diversion targets and address climate change impacts.

Introduction

Globally, around one third (≈1.3 billion tonnes) [1] of food is wasted, producing 8 to 10 per cent of the world's greenhouse gasses [2]. These gasses are known to contribute to a worldwide cycle of increased severe weather events, which then impact on food production, human health

and the environment [3]. Despite evidence of successful organics recycling systems, such as in South Korea, parts of Canada, the United States, and Europe [4], much of the worlds food waste is still sent to landfill. With predicted food waste per capita continuing to rise [5], households have been recognised as major contributors to household food waste (HFW) [6, 7]. This has generated a growing body of research addressing households' food waste production, with consumer behaviours targeted for food waste avoidance interventions, from addressing poor storage and use-by date adherence, to portion sizes and use of leftovers [8, 9]. Although individuals need to be held responsible for managing their own waste [10], Australian Local Governments (here referred to as councils) are responding to Federal and State/Territory food waste requirements (mandates and targets) by introducing systems to divert HFW away from landfill into an increasingly important, if still vestigial, circular economy [11, 12].

Europe provides working examples of how their councils divert HFW into a circular economy, such as energy production through anaerobic digestion [13] and soil improvement through composting [14]. Other diversion options are finding traction including food waste valorisation through insect processing [15] and biorefining [16], albeit that householder waste avoidance has been shown to be the greatest environmental and economic cost saving [11]. In seeking to address the 7.6 million tonnes of food it landfills annually [17] and engage stakeholders with 'cooperative interventions' [18], Australian State/Territory governments have favoured council implementation of a food organics/garden organics (FOGO) kerbside collections. Calls for reduction of HFW to landfill are not new [19] and, although FOGO uptake has been slow, shifts in political prioritisation of food waste is evident through the strategies [20], mandates and targets which have placed Australian councils at the forefront of HFW diversion [21]. As councils fall within the legislative frameworks of their States/Territories [22], the expectation is that they will enact their governing bodies requirements. Further encouragement is offered via substantial States and Territories grants, such as the New South Wales EPA organics infrastructure grants [23] and Western Australia's Better Bins funding [24]. Despite this increase in political will and funding availability, at the time of writing ~25% of Australian councils have implemented FOGO [25] (see Fig 1).

Fig 1 clearly demonstrates the geographic disparities in FOGO implementation. It also hints at socio-demographic and political differences, suggesting that those areas with better access to contractors, infrastructure, processors and markets appearing to be most able to respond to official targets and mandates. Whilst geographic location may be a defining factor for rural councils, it does not explain why many of the more urbanised councils have not implemented food waste diversion. Indeed, a brief desktop review of State/Territory [26] and councils waste management plans indicates that despite general support for HFW diversion from landfill, with some councils in process of implementing FOGO, a larger number are delaying. This suggests that, despite State/Territory extensive education and engagement programs with council waste management, there are unresolved issues around accepting FOGO as a suitable means of diverting food waste from landfill.

HFW diversion methodologies are causing a range of debates academically, industrially and politically [27]. On the one hand, FOGO is viewed as an essential component needed to 'lift recovery rates significantly' [28]. A recent feasibility report by the Food and Agribusiness Growth Centre [29] indicated that without a rapid increase in FOGO provision, the targets set out in the Australian National Food Waste Strategy of reducing HFW by half by 2030 [20] will not be met. Supporters of FOGO argue that failure to meet the targets will continue to allow HFW dumping in landfill and its associated greenhouse gas production [30], as well as damage the credibility of these targets within the community. However, caution has been advised when implementing the expensive and exacting system change and infrastructure development required by FOGO [31]. Additionally, FOGO has been identified as encouraging

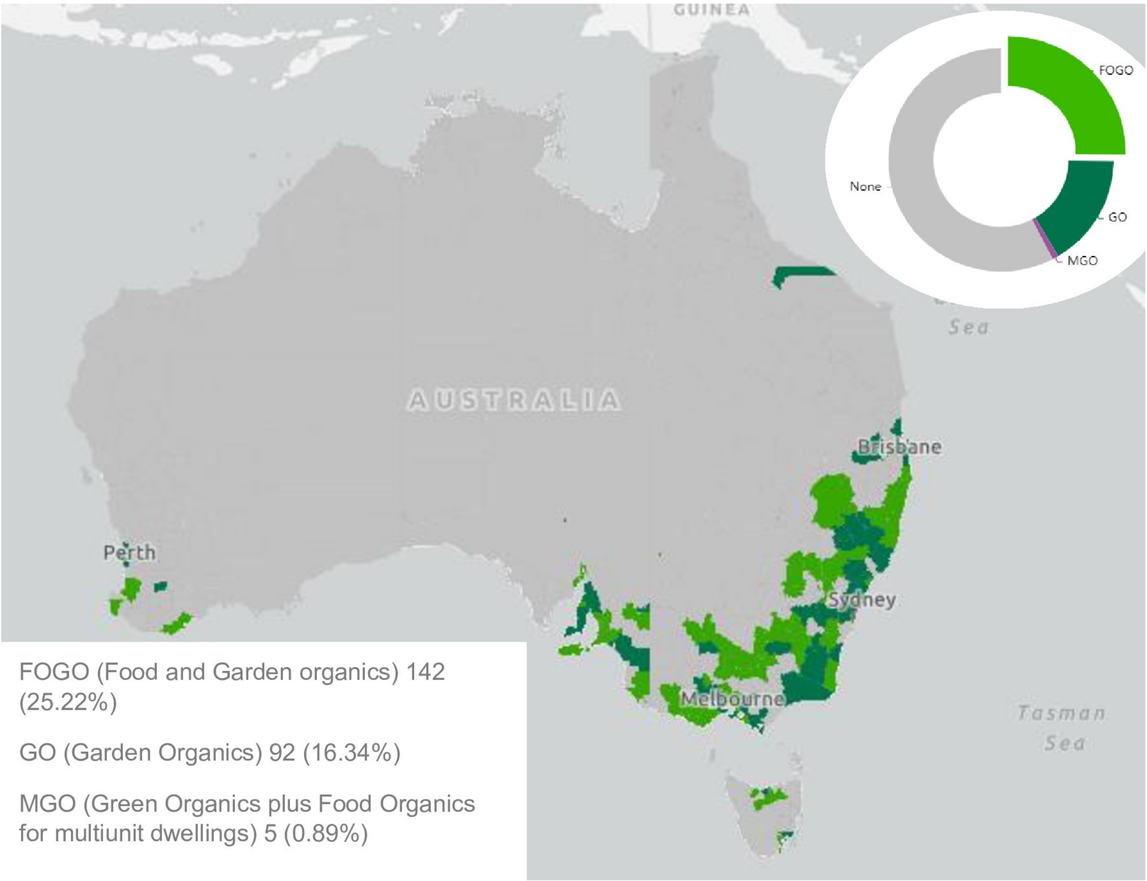

Fig 1. Tracking map showing FOGO and GO provision across Australia's 563 councils. Reproduced with permission from the NSW EPA.

https://doi.org/10.1371/journal.pone.0303391.g001

consumer abdication of responsibility at the cost of HFW avoidance [32, 33] thereby supporting a linear, as opposed to a circular, economy [34].

Responsibility in the context of this paper is associated with council-led HFW interventions. Without negating the importance of other key stakeholders (e.g. residents, businesses, contractors, and processors), when it comes to kerbside systems, council-based waste managers (CBWMs) are responsible for advising on and then delivering the service. Waste managers generally provide the reports to council that specify their considered best choice. This raises as yet unasked questions regarding where waste managers source their information, how this information is filtered according to their own attitudes and experience, and how this relates to their HFW decision making.

Australian CBWMs use a range of methods to acquire information, however it is not clear why the resulting decisions around HFW vary across apparently similar council situations, and whether factors such as confirmatory bias [35], or cognitive rigidity, influence FOGO implementations. To explore this, three objectives were applied. Firstly, to use validated measures to quantitively explore CBWMs HFW system preference in conjunction with their preferred sources of information and sense of structural empowerment. Drawing on these preferences, the second objective was to examine CBWMs sense of structural empowerment qualitatively via interviews. A research framework based on four pillars of structural

empowerment (resources, knowledge, support, professional development) and an attitudinal summary focusing on waste advocacy was used. The third objective was to integrate these results discursively to gain a better understanding of the interaction of CBWMs lived experience and attitudes. These objectives are reflected in the following research questions:

RQ1: **Knowledge**: By what means do waste services staff prefer to source household food waste related knowledge? Are these understandings filtered through a cognitive bias associated with waste managers preconceptions around council and community capacity?

RQ 2: **Resources**: what role do resources play in HFW decision making and do waste managers attitudes impact on their application?

RQ3: **Support and Advocacy**: Does having a sense of structural empowerment impact waste services staff with regards to HFW management and how does this respond to external factors?

Structural empowerment and waste management

Singh and Sarkar [36] argued that workplace innovation is dependent on organisationally supported psychological empowerment, known as Structural Empowerment [37]. With encouragement from Covid 19 and the stresses suffered in health services, the connections between organisational commitment with staff performance [38] and individual job satisfaction with knowledge sharing [39, 40] have gradually reached out to include other services, including waste management by councils [41]. One of the key components relates to job demands and resource availability, ameliorated by knowledge sharing opportunities and avenues. As pointed out by Kim and Lee [42], evaluating the interaction between these factors is not new [43, 44] and current studies on Australian councils continue to express lack of time, lack of resources and capacity as variables in effective waste management. Indeed, Bolger and Doyon [45] also highlighted interdepartmental knowledge sharing as a core component in achieving strategic sustainability goals.

Generic issues of 'resourcing, organisational capacity, supply constraints and cultural barriers' [46] are reflected in the risks associated with 'role stress' and decreased job satisfaction [40]. Job dissatisfaction and subsequent staff turnover in councils can potentially have significant impact on corporate waste services capacity and delivery through loss of knowledge. In 2022, the Australian Local Government Association [46] reported increased staff turnover within councils from 8.3% in 2018 to 15.6% in 2021, particularly in waste and water departments. It also identified that the adaptive capacity required for councils to respond to 'big picture' issues, such as climate change, pandemics and disasters, required skilled, well-resourced, and well-informed workers. Although the data does not show if staff move from council to council, it does indicate the importance of networking as a means of ameliorating job change, knowledge gaining and sharing.

Generally, research into FOGO implementation looks at the operational components and its environmental and financial costs [47, 48]. Few have explored council paradigms and their attitudes as a classification of type that affects system and policy choices. Drollery's 'minimalist, optimalist or maximalist' [49] classification encompasses councils' capacity and willingness to respond to change. Tran and Dollery [50] extend this into an argument against a 'one size fits all' policy approach as inherently flawed and based on incorrect assumptions of cost and community defining capacity.

Methods

Study design

In accordance with Cresswell (2014), a mixed methods research design involving a survey leading to follow-up interviews was adopted to gather experiences and rich insights from CBWM

staff across Australia. Informed by previous literature from related fields [51, 52], this approach was selected as the most appropriate method for engaging traditionally time poor participants. An online survey was designed to gather data and opinions from waste management staff across Australia. The survey data provided primary themes around perceptions of council, community and networks in relation to knowledge, self-determination, resources and support components of the SE model as shown in the results section. Although not an observational study, the STROBE checklist was used as a framework to ensure reporting consistency.

The semi-structured interviews drew on these themes whilst allowing participants discursive flexibility and providing opportunity for deeper exploration of the factors influencing household food waste management by councils. The Standards for Reporting Qualitative Research (SRQR) have been used to guide reporting of this study [53]

Sample and recruitment

The survey sample was drawn from CBWMs (including council-based managers, coordinators, project officers and educators) who responded to emails to Australia's 563 councils. An estimated minimum of 83 responses were required for sample validity (Australian Bureau of Statistics sample size calculator, confidence level 95%). Inclusion criteria were staff employed by council in a role involving waste management, over 21 years and able to understand complete a survey/interview in English. All Australian councils were contacted by email (n = 563) to identify staff responsible for waste services and establish if they were interested in contributing to the project. After follow-up emails to give more details and confirm their interest, those who were happy to contribute were provided with a research information sheet and a link to the Qualtrics survey which included the consent form. The survey was open for 3 months from May 20th, 2022. The survey questions were made available only after consent and eligibility were confirmed. At the end of the survey, participants were asked if they would like to take part in follow-up interviews and, if so, were directed to a separate survey to register participation and maintain anonymity of the survey. Interview participants were provided with a further information sheet, consent form and participant data card to complete and return before a time and date were selected. Interviews ran from August to November 2022. At the start of the interview, participants were asked if they had read the information had any further questions and were happy for the interview to be recorded. The study was summed up and participants were reminded that they could stop at any time. All participants were involved in council-led waste management from widely varying council situations across Australia. Basic demographics and roles are shown in Tables 1 and 2.

Data collection

Online survey. Developed with expert support (supervisors, statisticians), and distributed using Qualtrics [54], the survey was tested for face and content validity and refined before dissemination. Using Likert scales, yes/no answers and open-ended text responses, the survey questioned waste services staff's attitude and opinions towards their council's food waste interventions, their organisational sense of support and where they sourced their information and why (see Table 3). Responses were contextualised within each council's current HFW interventions and kerbside system, their means of community and in-council engagement with HFW interventions, and their levels of knowledge and perceived influence relating to HFW interventions.

Interviews. Drawing on four pillars of structural empowerment modelling (resources, knowledge, support and professional learning/advocacy), the interview protocol used a semi-structured approach to establish the rationale behind current waste management practices and

Table 1. Survey respondents base demographics (n = 163).

Survey Demographics		N
Gender	Male	70
	Female	91
	Prefer not to say	2
Age rage (years)	20–30	21
	31–40	41
	41–50	52
	51–60	39
	61 +	10
Time in role (years)	< 1	14
	1–5	77
	6–10	30
	11–15	18
	16 +	24

https://doi.org/10.1371/journal.pone.0303391.t001

opinions on future actions with regards to HFW. In keeping with research in the area [51, 52], a number of steps were involved in the development of the questionnaire for the semi-structured interviews in response to the themes drawn from the survey. An initial draft of the questionnaire was developed using themes from the survey as part of a professional interview training workshop (Best Practice Interviewing for Research Higher Degree Students, Griffith University) This determined the structure, flow, content, and delivery method, and helped define and test questions framed by the topic areas drawn from the survey. The questionnaire was then reviewed by supervisors (experts in food waste and psychology) and peers, and piloted by five CBWMs reflective of the target population to be interviewed. In keeping with the training, the most effective structure was found to be that which started with an initial technical question around the participants role and kerbside system followed by a series of reflective questions using both open and closed questions with verbal breadth and depth and non-verbal prompts. Responses were summarised back to participants to ensure understanding and avoid misinterpretation. Initial questions established the participants role and waste-related experience (‘*Tell me about your role and how you arrived at it*?’) and their council’s waste management systems (‘*What is your current kerbside system*?’) and thoughts on household food waste (‘*What do you think about household food waste*’ and ‘*How should household food waste be managed*?’). Levels of structural empowerment were explored through questions on working across departments, efficacy of interventions, connection with community, council and Councillors, and thoughts on future waste management by councils.

Table 2. Interview respondents base demographics (n = 45).

Interview Demographics		N
Gender	Male	18
	Female	27
Time in role (years)	< 1	4
	1–5	20
	6–10	6
	11–15	3
	16 +	5
	Prefer not to say	7

https://doi.org/10.1371/journal.pone.0303391.t002

Table 3. Sections of the survey relevant to this study.

Survey	Question and Choices	Purpose
SysOpn1 (Likert scale)	*In your opinion what rating would you give in general to the following household food waste management systems*? (1 star poor, 5 stars excellent)<br>SysOpn1_1 FOGO kerbside collection system<br>SysOpn1_2 Interventions that increase householder responsibility<br>SysOpn1_3 Alternative kerbside collections without any household food waste separation	Identify opinions around household food waste management systems and level of interest
Coms1 (Slider)	*How often do you discuss household food waste with anyone in your workplace*? (0 = never, 1 = once a week, 2 = 2–3 times a week, 3 = 4–6 times a week, 4 = daily)	
Coms2 (Likert scale)	*Who do you discuss household food waste with within council*? (never, rarely, sometimes, often, very often)<br>Coms2_1 Managers<br>Coms2_2 Councillors<br>Coms2_3 Waste team<br>Coms2_4 Other council departments	Identify networking within council
Coms3 (Likert scale)	*Who do you discuss household food waste with outside of council*? (never, rarely, sometimes, often, very often)<br>Coms3_1 With funding bodies<br>Coms3_2 With contractors<br>Coms3_3 With your residents	Identify networking outside of council
ComsSubj1 (text)	*In relation to household food waste, what are your most commonly discussed subjects*	
Comsb2 (text)	*Is there anyone else that you discuss household food waste with within council*?	
Comsb3 (text)	*Are there others outside of council that you discuss household food waste with*?	
Ntwk3 (Likert scale)	*Which sources do you as an individual person mostly get your waste-related information from*? (none, a little, a lot)<br>Ntwk3_1 Colleagues<br>Ntwk3_2 Conferences<br>Ntwk3_3 Government publications<br>Ntwk3_4 Industry meetings<br>Ntwk3_5 Internet sites<br>Ntwk3-6 LGA<br>Ntwk3_7 Research papers<br>Ntwk3-8 Waste associations<br>Ntwk3_9 Waste industry publications	Identify most common and preferred knowledge gaining pathways
Ntwk4 (text)	*Please outline any other additional sources of information that you find useful.*	

https://doi.org/10.1371/journal.pone.0303391.t003

Interviews and analysis were conducted by the lead author E Landells (EL) who has a background in council-based waste management and community engagement. Interview approach was constructed with support from the Griffith University Centre for Investigative Interviewing. As an insider researcher, reflective practice using annotations and a research journal were employed to ensure that the benefits of rapport and familiarity with the subject area did not create an imbalance of power or increased assumptions [55, 56] whilst allowing for an appreciation of the complexity of issues. Interviews were approximately 30 to 45 minutes and were transcribed using NVivo. EL knew three of the interviewees prior to the interviews. Regular meetings with co-authors were held to discuss the analysis and ensure a biased interpretation was avoided.

Ethics. Participation in the survey and interviews was voluntary and all information received was de-identified. No direct quotes were used from the survey participants however

interview participants are identified using Int-Pt followed by the appropriate number to maintain anonymity in accordance with ethical requirements. All materials and processes were run in accordance with the Australian National Statement on Ethical Conduct in Human Research and ethics approval was gained from CQ University's Human Ethics Committee (approval no. 0000022641). Consent was obtained from participants prior to participation.

Data analysis

Online surveys. The survey responses were analysed using SPSS and Excel. Descriptive explorations of the data (Pearson's Correlations) were used to measure the strength of linear relationships between the variables. Strong associations with correlations of ± 0.5 were retained. Reliability and consistency of the constructs was tested using Cronbach's alpha where.70 was recognised as good,.80 as better and.90 excellent. Factor analysis with oblique rotation was used to further clarify the variables and explore the underlying relationships between, in this case, participants sense of role empowerment (structural empowerment) and their preferred HFW knowledge sourcing methods. Text responses were analysed separately using Nvivo 12 to identify themes and patterns [57], with this process following the same three stage process (see Fig 2) as the interviews to ensure trustworthiness and rigour.

Interviews. In common with similar studies [58, 59] data was taken both from the open-ended survey questions and from the interviews and subjected to content analysis via NVivo 12 software and Excel. In line with Braun et al. [57] arguments on the benefits of online surveys for gathering qualitative information, thematic analysis of the text responses provided accessible points of entry for the interviews to assess current concerns around council-led household food waste interventions. In accordance with Charmaz's [60] line-by-line coding strategy and following Saldana's [61] advice on use of manual and CAQDAS coding (NVivo) to elicit a trustworthy analysis, the texts were systematically read in context and coded according to similarities with the aim or condensing the text, not reducing, and deducing themes [61]. To ensure correct interpretations, the transcripts were sent to participants for review of accuracy and completeness. After these were returned, to provide a rigorous and replicable approach, a codification process was created as per Roberts et al. [62] process (see Fig 2). This involved an overall summary of the text responses initially across the whole survey, and then expanded by

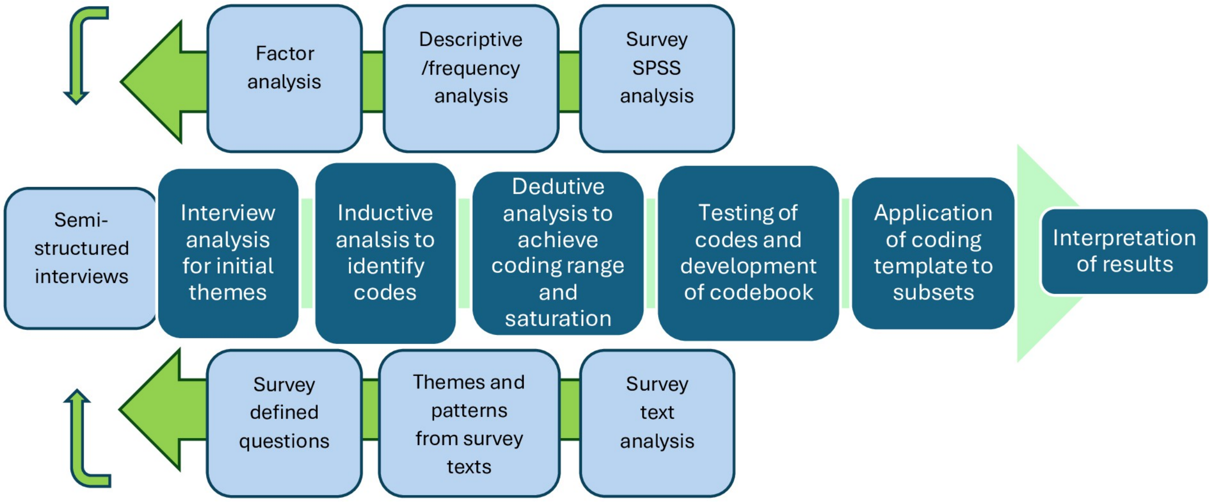

Fig 2. Data analysis sequence adapted from Roberts et al. (2019) showing the feed in of qualitative and quantitative data from the survey to the semi-structured interviews.

https://doi.org/10.1371/journal.pone.0303391.g002

Table 4. Results identifying most commonly used sources of information by council-based waste managers.

Ntwk3. Which sources do you as an individual person mostly get your waste-related information from?				
	Use a lot	Use a little	Do not use	Groupings
1. Ntwk3-8 Waste Associations	51%	41%	7%	Informal
2. Ntwk3-5 Internet Sites	51%	46%	2%	
3. Ntwk3-1 Colleagues	49%	40%	11%	
4. Ntwk3-3 Government Publications	41%	50%	9%	formal
5. Ntwk3-4 Industry Meetings	41%	43%	15%	
6. Ntwk3-6 LGA	39%	54%	7%	
7. Ntwk3-8 Waste Industry Publications	33%	59%	9%	
8. Ntwk3-2 Conferences	28%	52%	20%	
9. Ntwk3-7 Research Papers	12%	64%	24%	

https://doi.org/10.1371/journal.pone.0303391.t004

the interviews, into initial themes and subjecting the data to deductive (testing of assumptions/theory around waste management) and inductive (developing concepts) processes. This resulted in reviewable codes from which a codebook was developed. The codebook was then applied to each subset, re-reviewed to ensure breadth of coding and saturation, and used in this paper to provide cohesive results from which interpretations could be drawn.

Results

Survey findings

The survey results raised questions around knowledge sources and levels of structural empowerment and cognitive dissonance around HFW interventions. When asked about plans to change kerbside system to include HFW, 53 (29%) responded yes, 32 (18%) responded no and 97 (53%) did not answer. Relationships between CBWM staff and their preferred information sources are show in Table 4. A three-point Likert scale of 'use a lot', 'use a little' and 'do not use' provided the measure. Of the nine items assessed to identify preferences, three informal and 7 formal preferences emerged, headed by the informal group; 1) Waste Associations (51% use a lot) namely WMRR, the Waste Management and Resource Recovery Association of Australia. WMRR is recognised as Australia's peek body for the Australian waste industry and contains high levels of networking and information sharing as well as formal meetings, workshops and presentations. 2) Internet searches (51% 'use a lot'). 3) Colleagial networking (49% use a lot).

The formal grouping Government Publications and Industry meetings followed at 41%. Surprisingly, Local Government Associations listed 39%. The lower use of waste industry publications (33%) and conferences (28%) may confirm a temporal factor as these are not so regular or easily available as the internet and colleagues. Research papers were least used as preferred sources of information, rating highly as 'used a little' (64%) and the highest amongst 'do not use' (24%).

Table 5 showed clear loadings onto four components which were summarised under the following themes. 1. Structural empowerment by role—respondent's perceptions of support within their role, 2. Formal information (structured presentations, meetings and publications), 3. Structural empowerment by HFW opinion—structural empowerment in relation to HFW interventions and 4. Semi/informal information sources (colleagues, social media and LGA networks).

A total of 173 text responses were received from the survey regarding sources of knowledge. Thematic analysis categorised these into five information source groupings (formal, colleagial,

Table 5. Four factor model using Principal Component Analysis with Oblimin with Kaiser Normalisation rotation.

Variables		Factors				Tests	
		1. SE by role	2. Formal info. sources	3. SE by HFW opinion	4. Semi/informal info. sources	AVE	CA
Emp2	*When it comes to your role, do you feel. . .*						
Emp2_1	Your opinions are valued?	.900				.670	.921
Emp2_2	You have the scope to work effectively?	.885					
Emp2_3	Your decisions are supported?	.861					
Emp2_4	Your perspectives are understood?	.840					
Emp2_6	Supported introducing change?	.753					
Emp2_5	Encouraged to keep well informed?	.648					
Ntwk3	*Which sources do you [. . .] mostly get your waste-related information from?*						
Ntwk3_9	Waste Ind. Publications		.829			.491	(.814)
Ntwk3_8	Waste associations		.783				
Ntwk3_2	Conferences		.667				
Ntwk3_7	Research papers		.659				
Ntwk3_3	Gov. publications		.654				
Ntwk3_4	Industry meetings		.583				
Emp1	*When it comes to food waste management, in your opinion does your council. . .*						
Emp1_1	Encourage discussion with staff?			.860		.604	.893
Emp1_5	Take a waste-related leadership role in the community?			.810			
Emp1_2	Encourage innovative ideas?			.808			
Emp1_3	Support decision making?			.754			
Emp1_4	Understand domestic waste beyond the direct team?			.718			
Emp1_6	Dictate your priorities?			.701			
Ntwk3	*Which sources do you [. . .] mostly get your waste-related information from?*						
Ntwk3_1	Colleagues				-.751	.398	(.814)
Ntwk3_5	Internet sites				-.639		
Ntwk3_6	LGA networking				-.471		

SE = Structural Empowerment. CA = Cronbach's Alpha

https://doi.org/10.1371/journal.pone.0303391.t005

social media, economic and community), with colleagial communication the most frequently referred to (83 mentions) (see Table 6)

Thematic analysis of the 436 text responses received to the question about most commonly discussed HFW subjects identified change management and stakeholder engagement as the most commonly discussed topics (36%), followed by the logistics and issues with FOGO implementation (29%), with alternatives to FOGO least discussed (13%).

These results combined to form the guiding questions for the interviews.

Interview findings

43 waste services staff were interviewed, and the themes arising from the data were framed by the tenets of structural empowerment—resources, knowledge, support and professional development. Without negating conceptual overlaps, the main themes relating to this paper were:

1) Resources (structural, financial, informational): 1a: 'We haven't got resources to do what we've got to do'; 1b: 'It's not a one size fits all solution'; 1c: 'We are keen to see how the funding rolls out' and 1d: 'Well, why don't we just do it ourselves?' 2) Knowledge (formal and informal

Table 6. Groupings derived from thematic analysis of participants sources of knowledge text responses.

**Ntwk4:** ***Please outline any other additional sources of information that you find useful.***		**n**	**%**
Semi-formal and Colleagial networks	Knowledge sourcing and sharing:<br>Colleagial and council networks, waste and waste educator networks, colleagues from other councils, networking groups, tours and visits, team talks, toolbox meetings<br>Other council departments	83	48%
Formal networks	Operational sources, LGAs, regional waste groups, regional organisation of councils (ROCs), waste management authorities, EPAs<br>Knowledge building—Publications, specific online sources, articles and reports	33	19%
Economic networks	Market development, market capacity, resources, negotiation, commercial networks, contractors, industry contacts, manufacturers	28	16%
Social media networks	Online sources, social media, google, LinkedIn, Instagram, Facebook, council websites, apps	15	9%
Community	Community, focus groups, schools, community organisations, Councillors	14	8%

https://doi.org/10.1371/journal.pone.0303391.t006

sources): 2a: 'Our responsibility to kind of keep up'; 2b: 'Networking opportunities are definitely key'; and 2c: 'Get the benefit of the other knowledge in the room.' 3) Support (internal and external): 3a: 'Very supportive, very environmentally focused and knowledgeable'; 3b: 'Lots of internal support from staff as well'; 3c: 'We had garden waste bins thrown at the Civic Centre building'; and 3d: 'Are you guys having the same issues we are?' 4) Professional development (HFW advocacy): 4a: 'We want to have a lot of things in place before then'; 4b: 'Just try to go to as many webinars'; 4c: 'We're on that journey'; and 4d. 'I don't know that that's going to happen.'

Resources

We haven't got resources to do what we've got to do. As a pillar of structural empowerment, resources combine structural, goal orientated mechanisms [63] with Bandurian psychological constructs [64], the lack of which allows promising ideas to 'die down' [65]. Participants identified resource constraints as a major factor in applying HFW interventions ('. . .being a small council, we don't have a lot of resources, which makes it very challenging.' Int-Pt-19). Typically, issues around organisational or locational barriers were raised, such as lack of staff, lack of time, lack of physical resources and logistical issues:

> *"My problem is in a small council is I'm completely under-resourced. So, I'm, you know, I can go from picking FOGO, so I'm the manager, and I can go and pick FOGO on Monday to high level strategy meetings in the same hour. Really, so time is the issue for me."*
>
> *Int-Pt-10*

It's not a one size fits all. Despite the accusation of Australia's 'soft law' [66] approach to HFW management, participants without FOGO often referred to a common set of arguments regarding HFW targets and mandates as unrealistic for their specific situations, although under pressure to implement ('. . .and we're going, 'No, we don't want to be involved.' You know, we haven't got resources to do what we've got to do, never mind take something else on.' Int-Pt-9), highlighting council diversity:

". . .advice to the various levels of government that that funding is required to get these projects going and it's not a one size fits all solution. That's probably the biggest one, particularly with the standardised everyone to have a FOGO bin. I know it's got some certain exceptions, but it's very ambiguous on what those exceptions are. [. . .] there's also examples where a FOGO bin isn't really practical. . ."

Int-Pt-6

We are keen to see how the funding rolls out. In common with the United States and New Zealand [67], Australian States/Territories are in the process of determining what infrastructure is required and where to process organics [68]. Participants repeatedly mentioned funding associated with FOGO related infrastructure development.

"There was some good funding announced a while back jointly between Federal, State and the councils, in the sort of billions of dollars sort of numbers. [. . .] not all for waste, but a lot of money for infrastructure and waste management services in this area. So, we certainly are keen to see if there's some funding opportunities for FOGO as well. It's a little bit of a new game for Queensland. And so, we are keen to see how the funding rolls out. . . ."

Int-Pt-12

Well, why don't we just do it ourselves?. Where waste managers attitudes, capacity to innovate and internal support systems aligned, the cognitive dissonance created by HFW requirements resulted in practical solutions. Where demonstrated, this supported the concepts of resource orientated cognitive convergence motivated by social learning and innovation [69, 70]. Where pro-active CBWMs were 'sick of waiting' (Int-Pt-22), localised solutions were sort:

"Transport was going to be the killer. If I had added transport to it, it just made the program non-viable. So, we just said, 'Well, why don't we just do it ourselves?' It can't be that hard. So, we trialled it. We did it and we've actually produced a really good product. So, we're not really interested in playing in the commercial composting space at the moment. We're just managing our own FOGO on our own aerated floor static pile. We're not windrow turning, not creating any odour. . ."

Int-Pt-10

Knowledge (formal and informal sources) interpretation and application

Participants were asked about their experience in council-based waste management, their opinions on HFW and how they sourced their knowledge. Responses appeared to be filtered through attitudes and council-specific factors ranging from proactive/driven—'So we are now celebrating 10 years of having our Fogo service in place' (Int-Pt-28), and 'branded the sustainable Games [Olympics]' (Int-Pt-12), observing ('we're just kind of sitting back waiting to see what our adjoining councils do.' Int-Pt-2) and constrained/resistant ('I'm not going to get people to do this [FOGO] right if we're dragging them kicking and screaming.' Int-Pt-34).

Our responsibility to kind of keep up. In common with the survey, a relatively small set of formal and informal knowledge sources were mentioned, with Government (State/Territory) and the peek industry group WMRR recognised as primary initial information sources

and networks/networking groups as a means of interpreting information and assessing options, summed up here as 'I guess federal and state legislation kind of just cascades down to the local level, and it's really our responsibility to kind of keep up with that.' (Int-Pt-17). Many participants commented on WMRR as both an important information source and a networking opportunity.

"WMRR is a really, really good resource for across Australia. I think if we if I didn't have that, I would probably only have that view of what's happening in WA, and you probably wouldn't necessarily see what's happening over East."

Int-Pt-7

Networking opportunities are definitely key. Participants unanimously stressed the benefits of networking as a means of gaining and sharing knowledge ('So, it's networking [. . .] like 'has anyone done this' and sharing of resources, sharing our ideas, experiences, things to be aware of and stuff like that.' Int-Pt-1) in what they recognised as a rapidly changing HFW paradigm [71]. This included internal and external knowledge sources and highlighted a need for availability of knowledge derived from experience and those councils and individuals in similar situations:

". . .I find the WMRR group is really good. So, I joined the WMRR young professionals as well just to try and make some new networks and I still talk to all my colleagues in WA and in Victoria as well. And just on those networking opportunities is definitely key and just talking to people in the industry. . ."

Int-Pt-4

Formal and informal groups were appreciated for networking opportunities.

"We've had WENG, which is the waste education network is a group [. . .]. We've also got the Consistent Communications Collective, which is run by WALGA."

Int-Pt-16

Get the benefit of the other knowledge in the room. The interviews elucidated a sense of cohesion between with the relatively small number of waste management professionals in Australia, a distinct willingness to share information and expertise, plus a reliance on their experience:

"We've had people that have worked in my team that worked for Veolia, for Cleanaway. They've got years of experience and with that comes with contacts as well. So, we always have someone that we can ring up if needed."

Int-Pt-8

As well as reading, the value of networks cannot be overstated, for both old and new ventures where networks tell of '. . .the latest changes that are coming through policy-wise or, um, you know, new opportunities coming out of things that the State or whoever is doing.' Int-Pt-12). Frequently, participants commented on their resource constraints. This connected with

them seeking means of overcoming these by connecting with those in similar situations, such as 'trying to get the benefit of what somebody is struggling with. Get the benefit of the other knowledge in the room.' (Int-Pt-3) and:

> *"Being part of a regional group is that of connecting with your neighbouring, particularly neighbouring, councils or people who have a similar situation to you and knowing who to call."*
>
> *Int-Pt-3*

Support. Whilst HFW related knowledge was externally sources, participants talked about internal council processes. managerial hierarchies and councillor approvals as supports for decision making.

Very supportive, very environmentally focused and knowledgeable. In talking about the high level of support required for introducing a FOGO system, the risk averse nature of waste managers and the need to use process to ensure political and executive level support became clear. Whilst appreciating that 'politicians [. . .] are able to really steer a lot of good programs' (Int-Pt-10), internal support was often referred to:

> *"Waste is very expensive, so that gets the attention of higher management and our Councillors because it's really important that it's done properly and cost effectively. So, it depends on what council we get to as to how environmentally focused they are, and what CEO we have. So, it seems to change a little bit. But in terms of direct management, very supportive, very environmentally focused and knowledgeable to not just someone who doesn't know anything about it."*
>
> *Int-Pt-2*

Lots of internal support from staff as well. Once prompted, participants noted the need for inter-departmental support mostly from those involved with introducing change, such as community engagement and communications departments.

> *"Our council knows that our landfill is drawing to a close. And so, it's important for now and whatever happens in the future to take that [HFW] out of the landfill. [. . .] So, we had a lot of support from council and yeah, lots of internal support from staff as well."*
>
> *Int-Pt-45*

We had garden waste bins thrown at the civic centre building. Some participants commented on the need for consistent and ongoing organisational support as an essential component in achieving effective community engagement with kerbside system change. Waste management plans, strategies and reports were commonly referred to as the underpinnings to, and means of entrenching, awareness raising within and by council.

> *"We've just got to ride through the wave of pain to get there. And that will happen, for example, when we introduced compulsory garden waste about 10 years ago, we had protests. We had garden waste bins thrown at the Civic Centre building. [. . .] So, it's about really the ability to plan to be prepared for the backlash that will happen and have enough support in the organization to get through that.*

Int-Pt-34

One participant mentioned lack of firm State legislation in Western Australia as a disincentive for FOGO introduction, as well as perceived issues with support for market development.

"The Waste Strategy is not mandatory. As far as I'm aware, which is probably kind of the lever that a lot of councils are using not to go FOGO at this point because there isn't a firm line in the sand saying that you have to."

Int-Pt-11

"I know the regional councils like Bunbury is having quite an issue in terms of actually having someone to take the end product and to do the processing. So that's. . .again, one of the things that the councils who are holding out pulled up to say, 'Oh, there's not a guaranteed end market, we don't want to make this move.' So yeah, and it will be interesting to see how the Waste Authority then manages that going forward."

Int-Pt-11

Are you guys having the same issues we are?. Whilst internal channels provided process and decision-making support, participants made the importance of external support for information and options very clear.

"And that's something that I've noticed is when I was in the private industry, it was very much 'this is our IP, we don't share this.' And then when I moved into government and things like that it was 'share it with everyone, they'll love it.' It's just fantastic because [. . .] we're all trying to achieve the same thing."

Int-Pt-2

Advocacy (professional development) for HFW

We want to have a lot of things in place before then. Waste managers HFW perspectives determined the types of information they required with participants in the process of introducing FOGO the most motivated to seek solutions including validating technology. High level external drivers placed these participants in more immediate situations.

"And our region here, we're part of the um, the southeast Queensland area that's all part of the 2032 Olympics. So, um, you know, we will be part of the focus there, and that's very much, you know, going to be branded the sustainable games, and I'm sure, you know, we want to have a lot of things in place before then."

Int-Pt-12

Those with less pro-active or less influential motivators (observing and constrained) talked about inspections of technology and the practical insights gained from other council's implementations.

"I like to have a go and actually see it in operation somewhere and that, you know, it's always. . .because people can write up a lot of articles and say how great things are they. . . sometimes you go and visit, and it might not be exactly all roses."

Int-Pt-18

"We're waiting for those guys to get it up and running [commercial infrastructure] and doing it perfectly in a sense, so we could then take that on."

Int-Pt-29

Just try to go to as many webinars. Post Covid, the availability of webinars has continued to be a named source of learning for waste managers.

"Just basically kind of subscribe to anything that going and sitting on webinars and yeah, try and really keep a finger on the pulse and read news articles and industry newsletters."

Int-Pt-45

We're on that journey. Solutions-focused professional development was highly valued ('Learning from other councils and kind of, you know, working out what's been done' (Int-Pt-17). However, it exposed issues that many of the non-FOGO councils reported struggling with:

"We're kind of now playing catch up. And that's fine but it also means that we've probably got quite a bit of investment ahead of us to get us to the skill level where a lot of the other council's already are. . . and it has to happen. And, you know, we're on that journey. But yeah, it means [. . .] we don't have the ability to respond to things like, you know, the food waste initiatives stuff as easily as some of those. I don't know what the solution to that is with the resources that we have."

Int-Pt-24A

I don't know that that's going to happen. One regional coordinator expressed frustration with a lack of advocacy for HFW within councils.

"Where I'd like it to go is where the councils properly resourced their waste team because at the moment, there's all of this funding that's going to become available. They're not going to be able to get any of it because they're scratching their tails, just trying to react to issues. So, I think if somebody could convince councils that funding waste is going to pay for itself then that would make a really big difference, but I don't know that that's going to happen."

Int-Pt-3

Summary of structural empowerment and CBWMs influence

We've told them. FOGO pro-active participants talked about the importance of FOGO related groups, meetings and discussion, such as the Western Australia Consistent Communications meetings, the FOGO Reference Group, colleagues in a 'similar situation' (Int-Pt-3), and the importance of information on what the FOGO processors will take (Int-Pt-11). Participants from the 'observing' councils talked about 'waiting for those guys to get it up and running' (Int-Pt-29) and wanting to see how the others did it first—'we're just kind of sitting back waiting to see what our adjoining councils do' (Int-Pt-4). Indicators of waste managers influence is as evident here as with the other two groups.

"Councillors ask about it [FOGO] because it's the buzz topic at the moment. But we've told them, we left them in no uncertain terms, that we need to start educating our community at least 12 months before we introduce it, because otherwise it's just another bin. [. . .] they have to put out on the kerb."

Int-Pt-41

Have we messaged anyone on waste?. The majority of participants recounted community and council connection as a 'journey' they were undertaking. For the extremely resource constrained councils, such as those rural, regional and small councils, who struggle with implementing any form of waste management system, kerbside collections, let along HFW management, had yet to reach the agenda:

"Ask me, have we messaged anyone on waste? Well, I don't believe we have. No!"

Int-Pt-13

They could be known as. . . Dependency on external resourcing under-shadowed many of the responses, as well as providing reasons for inaction by observing, less pro-active councils. This does not mean that constrained councils lacked the commitment to achieve HFW outcomes, should they actively attract funding.

". . .if they'd set it up right now, they could be known as the people that set, like, set Central Australia up for the future rather than just left it till technology caught up"

Int-Pt-29

One participant noted an information and networks constraint due to location.

". . .cos we haven't been to New South Wales or Queensland, you haven't got that contact, so you haven't got any information unless it's coming through an industry body like Waste Management Review"

Int-Pt-29

Discussion

Using four pillars of Structural Empowerment as a framework, this study sequentially investigated how CBWMs interact with decision making around HFW interventions—primarily from the perspective of HFW diversion from landfill via FOGO. Thematic analyses identified attitudinal divisions between the use of formal and informal knowledge sources contextualised by CBWMs available resources, current knowledge and perceived capacity of their council and community. Whilst the data indicated levels of cognitive cohesion across the CBWM group in relation to waste management, and identified similar barriers to HFW intervention implementations, it did not show cohesion in approach. Indeed, the data highlighted a series of practical implications for HFW diversion drawn from a significant knowledge gap between identification of a council's needs in conjunction with its paradigm (political and governance) and the waste managers attitudes, together with flexible solutions for HFW interventions.

Structural empowerment is defined as a means of 'mobilising resources, achieving goals through perceived and real levels of support, resource accessibility and opportunity [72]. It

therefore extends beyond role satisfaction into interpretation of the waste services situation within councils and participants level of contentment with this. Participants responses indicated that both sourcing and use of information depended on a combination of factors, primarily individual and organisational attitudes, perceptions of capacity and agendas. Individual councils paradigm and type (here defined as pro-active, observing or constrained) affected approaches to HFW [50] with the attitude and influence of the waste services team both resulting from, and contributing to, this discourse.

Measuring the attitudes towards real and perceived needs of a council's waste services would explain why some resource constrained councils are pro-active in the HFW field whereas others, with similar or more resources, are not. For observing-but-not-engaging councils, evidence supported regional policy interactions for overcoming resource issues and supporting waste services confidence in project implementation and system alignment [73]. As well as councils recycling systems benefiting from 'combination policies' [74], regional approaches emerged as means of sharing of information with those in similar situations, hearing about innovation, exploring localisation, and determining methods of overcoming similar issues. Where mandates require councils to divert HFW away from landfill, a format for assessing implementation methods that includes a needs assessment would be highly beneficial.

Set against their current kerbside systems, participants identified change management and stakeholder engagement as primary concerns, followed by how to implement FOGO. In common with recycling practices in households, attitude and ease of application are determining factor in behaviour change [34, 45] and, although the motivations are clearly different, CBWMs attitude, sense of organisational 'fit', and levels of influence affected their system choice. Indeed, participants exhibited attitude as both barriers and enablers, according to their opinions on FOGO, thereby categorising their sense of structural empowerment according to their organisational paradigm. If non-FOGO CBWMs felt their organisation was operating at the desired level with the correct amount of support for waste services, they would argue against system change. At a practical level, cognitive dissonance was not sufficient to induce change planning or innovation in resource application.

Without ignoring the holistic nature of knowledge gathering and the importance of the other sources identified here, participants use of informal avenues of networking, the internet and WMRR (as the peek body across Australia's waste industry) to interpret information suggests searches aligned to individual attitudes and/or organisation agendas. Taking social media as an example, despite the complexity councils find in adopting and managing the internet for community engagement [75, 76] there is little doubt that it is an important source of knowledge acquisition and dissemination. However, just over half of the survey participants rated the internet as used a lot for information sourcing. The interviews drew out the multiple roles of social media—to receive and search for information, to disseminate information to the community/network and to receive comment from the community. The point being that not only are informal and formal sources of information equally valid in a 'spectrum of approaches' [77], but also that attitude and influence need to align with policy and agenda for HFW targets to be met [78, 79].

The next stage is to explore the pragmatics of how to better engage less pro-active (observing) councils and overcome resource constraints. For example, where States and Territories have produced materials to support policy implementation, such as the NSW EPA *Scrap Together* [80] and Victoria's *Path to Half* [81], councils have generally adapted and rebranded them according to their own style guides. A quick review of council waste-related web pages, supported by data from the interviews, shows this variety clearly and confirms councils enduring preference for re-branding of external information. Frustration was shown by regional level participants who viewed this practice as wasting time and reinventing the wheel, a form

of 'territorial marketing' [82] and lack of standardisation as a failure in in effective urban planning. Whilst not negating the high levels of inter-regional cooperation between councils, council individualisation hints at differences in governance, leadership and innovative agility. More work needs to be done to better understand council's interactions with innovation on the one hand and standardisation on the other.

Conclusion

Results of this study showed a need to support council-based waste managers more effectively if Australia is to meet its household food waste diversion from landfill targets. The allocation of responsibility for HFW avoidance and diversion have primarily focused on consumers [83, 84]. To the authors knowledge, there is very limited current literature [85, 86] on how waste managers attitudes influence councils HFW interventions, yet their attitude is a key component in defining household food waste interventions.

The recommendations from this research are therefore:

1. **Expand successful models** through formal and informal networks to increase normalisation of HFW interventions. Waste management respondents in this study showed extensive depth of industry-related knowledge and experience and a willingness to share. As HFW interventions are set in the context of attitudes and perceived capacity, the availability of examples and deliberate mentoring by those in similar situations would be a strong benefit.
2. **Increase motivation**: Provide unambiguous political support e.g., re-evaluate the funding model to provide stronger support for resource constrained councils e.g., research needs to be done to assess the impact of changing to a needs assessed award across States/Territories rather than grants.
3. **Increase capacity and ease of access** e.g., design a solutions-focused assessment toolkit that provides both an analysis methodology and a selection of resulting pathways towards household food waste diversion strategies for councils to adapt according to their situations.

This paper has used an investigation of where waste services staff source information as a starting point for better understanding the factors influencing council led HFW interventions in Australia. It identified formal and informal pathways as part of a holistic communication base and has opened up many further avenues of enquiry. Most current of these, given the drive for diverting HFW food waste from landfill, would be further exploration of levels of influence that waste services have on council based HFW interventions. Also, the interaction of key stakeholders in council and the community, including local elected members, would provide more insights on waste management in Australia. Waste services teams, or those responsible for waste services delivery, are the fulcrum upon with council-based household food waste interventions depend. They are therefore a vital component in meeting Australia's food waste reduction targets, deserving of further exploration.

Limitations

This study has targeted what waste services staff need to know, not how they need to know. As such, it is only a starting point. Given recent recognition of pervasive toxins such as PFAS in compostable food containers and cardboard, the methods for handling post collection HFW need urgent review to ensure the intended environmental benefits are achieved [87]. It has taken the perspective that HFW diversion from landfill is a pro-environmental activity, but one that needs considerably more attention.

Acknowledgments

We am grateful to participants in both studies for sharing their time and expertise. I also acknowledge David H. Pearson from CQUniversity and the End Food Waste CRC for high-level supervision and taking carriage of important administrative tasks on behalf of End Food Waste CRC.

Author Contributions

Conceptualization: Esther Landells.

Data curation: Esther Landells.

Formal analysis: Esther Landells.

Investigation: Esther Landells.

Methodology: Esther Landells.

Supervision: Gamithri G. Karunasena, Samuel Oakden, Anjum Naweed.

Writing – original draft: Esther Landells.

Writing – review & editing: Esther Landells, Gamithri G. Karunasena, Samuel Oakden, Anjum Naweed.

References

1. Flanagan K, Robertson K, Hanson C. Reducing Food Loss and Waste: Setting a Global Action Agenda. World Resources Institute. 2019. https://doi.org/10.46830/WRIRPT.18.00130
2. UNEP. UNEP (United Nations Environmental Programme) Food Waste Index Report 2021. Nairobi; 2021. https://www.unep.org/resources/report/unep-food-waste-index-report-2021
3. FAO, IFAD, UNICEF, WFP, WHO. The State of Food Security and Nutrition in the World 2021. Transforming food systems for food security, improved nutrition and affordable healthy diets for all. FAO. Rome: FAO, IFAD, UNICEF, WFP and WHO; 2021 Jul.
4. Ebenezer AV, Kumar MD, Kavitha S, Uan DK, Banu JR. State of the art of food waste management in various countries. Food Waste to Valuable Resources: Applications and Management. 2020; 299–323. https://doi.org/10.1016/B978-0-12-818353-3.00014-6
5. Lopez Barrera E, Hertel T. Global food waste across the income spectrum: Implications for food prices, production and resource use. Food Policy. 2021; 98: 101874. https://doi.org/10.1016/J.FOODPOL.2020.101874
6. Principato L, Mattia G, Di Leo A, Pratesi CA. The household wasteful behaviour framework: A systematic review of consumer food waste. Industrial Marketing Management. 2021; 93: 641–649. https://doi.org/10.1016/j.indmarman.2020.07.010
7. Limon MR, Villarino CBJ. Knowledge, attitudes and practices on household food waste: Bases for formulation of a recycling system. Global Journal of Environmental Science and Management. 2020; 6: 323–340. https://doi.org/10.22034/gjesm.2020.03.04
8. Reynolds C, Goucher L, Quested T, Bromley S. Consumption-stage food waste reduction interventions —What works and how to design better interventions. Food Policy. 2019; 83: 7–27. Available: https://www.sciencedirect.com/science/article/pii/S030691921830318X
9. Hebrok M, Heidenstrøm N. Contextualising food waste prevention—Decisive moments within everyday practices. J Clean Prod. 2019; 210: 1435–1448. https://doi.org/10.1016/j.jclepro.2018.11.141
10. van der Werf P, Seabrook JA, Gilliland JA. Food for naught: using the theory of planned behaviour to better understand household food wasting behaviour. The Canadian Geographer. 2019; 63: 1–16. https://doi.org/10.1111/cag.12519
11. Slorach P, Jeswani HK, Cuéllar-Franca R, Azapagic A. Environmental and economic implications of recovering resources from food waste in a circular economy. Science of the Total Environment. 2019;693. https://doi.org/10.1016/j.scitotenv.2019.07.322 PMID: 31635000

12. de Sadeleer I, Brattebø H, Callewaert P. Waste prevention, energy recovery or recycling—Directions for household food waste management in light of circular economy policy. Resour Conserv Recycl. 2020; 160: 104908. https://doi.org/10.1016/j.resconrec.2020.104908

13. Loizia P, Neofytou N, Zorpas AA. The concept of circular economy strategy in food waste management for the optimization of energy production through anaerobic digestion. Environmental Science and Pollution Research. 2019; 26: 14766–14773. https://doi.org/10.1007/s11356-018-3519-4 PMID: 30377970

14. Ayilara M, Olanrewaju O, Babalola O, Odeyemi O. Waste Management through Composting: Challenges and Potentials. Sustainability. 2020; 12: 4456. https://doi.org/10.3390/su12114456

15. Ojha S, Bußler S, Schlüter OK. Food waste valorisation and circular economy concepts in insect production and processing. Waste Management. 2020; 118: 600–609. https://doi.org/10.1016/j.wasman.2020.09.010 PMID: 33010691

16. Jones R, Speight R, Blinco J, O'Hara I. Biorefining within food loss and waste frameworks: A review. Renewable and Sustainable Energy Reviews. Elsevier Ltd; 2022. https://doi.org/10.1016/j.rser.2021.111781

17. FAO. SDG 12. Responsible consumption and production | Sustainable Development Goals | Food and Agriculture Organization of the United Nations. 2021 [cited 24 Feb 2022]. https://www.fao.org/sustainable-development-goals/goals/goal-12/en/

18. Ghinoi S, Silvestri F, Steiner B. Toward the creation of novel food waste management systems: A network approach. J Clean Prod. 2020; 246. https://doi.org/10.1016/j.jclepro.2019.118987

19. Dawkins E, Allan P. Department of Sustainability, Environment, Water, Population and Communities Landfill Ban Investigation. 2010.

20. Commonwealth of Australia. National Food Waste Strategy: Halving Australia's food waste by 2030. Australian Government. 2017. https://www.dcceew.gov.au/sites/default/files/documents/national-food-waste-strategy.pdf

21. Herron M, Jones DS, Roös PB, Allam Z. Creating revenue out of green waste: New perspectives for municipal organic waste harvesting in Geelong, Australia. Geography, Environment, Sustainability. 2021; 14: 91–105. https://doi.org/10.24057/2071-9388-2020-182

22. Aulich C. Australia: Challenging institutional constraints. The Routledge Handbook of International Local Government. 2018; 363–376.

23. NSW EPA. Organics funding. 2023 [cited 17 Mar 2023]. https://www.epa.nsw.gov.au/working-together/grants/organics-infrastructure-fund

24. WALGA. Better Bins | Waste Authority WA. 2023 [cited 17 Mar 2023]. https://www.wasteauthority.wa.gov.au/programs/view/better-bins-program

25. Australian Government. Food Organics and Garden Organics Pickup Website. In: Dept of Climate Change, Energy, the Environment and Waster [Internet]. 2022 [cited 22 Oct 2022]. https://experience.arcgis.com/experience/e6b5c78e1dac47f88e7e475ffacfc49b

26. Queensland Government. Queensland Organics Strategy 2022–2032. 2022. file:///C:/Users/Esther/Downloads/organics-strategy-2022-2032.pdf

27. Jones S. Establishing political priority for regulatory interventions in waste management in Australia. Aust J Polit Sci. 2020; 55: 211–227. https://doi.org/10.1080/10361146.2019.1706721

28. Infrastructure Victoria. Recycling and resource recovery infrastructure: evidence based report. 2019 Oct. chrome-extension://efaidnbmnnnibpcajpcglclefindmkaj/https://apo.org.au/sites/default/files/resource-files/2019-10/apo-nid264221.pdf

29. FIAL. National Food Waste Strategy Feasibility Study Can we halve Australia's food waste by 2030? 2021.

30. Blair J, Mataraarachchi S. A review of landfills, waste and the nearly forgotten nexus with climate change. Environments—MDPI. 2021; 8: 73. https://doi.org/10.3390/environments8080073

31. Edwards J, Othman M, Burn S, Crossin E. Energy and time modelling of kerbside waste collection: Changes incurred when adding source separated food waste. Waste Management. 2016; 56: 454–465. https://doi.org/10.1016/j.wasman.2016.06.033 PMID: 27396681

32. Karunasena GG, Pearson D. Food Waste in Australian households: evidence for designing interventions. Adelaide; 2022. https://fightfoodwastecrc.com.au/wp-content/uploads/2022/04/Food-waste-in-Australian-households-final.pdf

33. Haque A, Karunasena GGG, Pearson D. Household food waste and pathways to responsible consumer behaviour: evidence from Australia. British Food Journal. 2021;ahead-of-p. https://doi.org/10.1108/bfj-05-2021-0517

34. Mak TMW, Xiong X, Tsang DCW, Yu IKM, Poon CS. Sustainable food waste management towards circular bioeconomy: Policy review, limitations and opportunities. Bioresource Technology. 2020. https://doi.org/10.1016/j.biortech.2019.122497 PMID: 31818718

35. Rabin M, Schrag JL. First Impressions Matter: A Model of Confirmatory Bias. Q J Econ. 1999; 114: 37–82. https://doi.org/10.1162/003355399555945

36. Singh M, Sarkar A. Role of psychological empowerment in the relationship between structural empowerment and innovative behavior. Management Research Review. 2019; 42: 521–538. https://doi.org/10.1108/MRR-04-2018-0158/FULL/XML

37. Kanter RMoss. Men and women of the corporation. New York: Basic Books; 1993. https://books.google.com/books/about/Men_and_Women_of_the_Corporation.html?id=B-5GztW4IZQC

38. Fragkos KC, Makrykosta P, Frangos CC. Structural empowerment is a strong predictor of organizational commitment in nurses: A systematic review and meta-analysis. J Adv Nurs. 2020; 76: 939–962. https://doi.org/10.1111/jan.14289 PMID: 31820471

39. Moura LN, Camponogara S, dos Santos JLG, Gasparino RC, da Silva RM, Freitas EDO. Structural empowerment of nurses in the hospital setting. Rev Lat Am Enfermagem. 2020; 28: e3373. https://doi.org/10.1590/1518-8345.3915.3373 PMID: 33174992

40. Orgambídez A, Almeida H. Exploring the link between structural empowerment and job satisfaction through the mediating effect of role stress: A cross-sectional questionnaire study. Int J Nurs Stud. 2020; 109. https://doi.org/10.1016/J.IJNURSTU.2020.103672 PMID: 32569828

41. García-Juan B, Escrig-Tena AB, Roca-Puig V. Structural empowerment and organisational performance: the mediating role of employees' well-being in Spanish local governments. 2020 [cited 6 Feb 2023]. https://doi.org/10.1080/09585192.2020.1859581

42. Kim S, Wang J. The Role of Job Demands—Resources (JDR) between Service Workers' Emotional Labor and Burnout: New Directions for Labor Policy at Local Government. Int J Environ Res Public Health. 2018; 15: 2894. https://doi.org/10.3390/ijerph15122894 PMID: 30562995

43. Lee RT, Ashforth BE. A meta-analytic examination of the correlates of the three dimensions of job burnout. Journal of Applied Psychology. 1996; 123–133. Available: https://psycnet.apa.org/buy/1996-00291-001 https://doi.org/10.1037/0021-9010.81.2.123 PMID: 8603909

44. Bakker A, Demerouti E, de Boer E, Schaufeli w G. Job demands and job resources as predictors of absence duration and frequency. Journal of Vocational Behaviour. 2003; 62: 341–356. Available: https://www.sciencedirect.com/science/article/pii/S0001879102000301

45. Bolger K, Doyon A. Circular cities: exploring local government strategies to facilitate a circular economy. European Planning Studies. 2019; 27: 2184–2205. https://doi.org/10.1080/09654313.2019.1642854

46. ALGA. 2022 Local Government Workforce Skills and Capability Survey Final Report. 2022.

47. Edwards J, Othman M, Crossin E, Burn S. Life cycle assessment to compare the environmental impact of seven contemporary food waste management systems. Bioresour Technol. 2018; 248: 156–173. https://doi.org/10.1016/j.biortech.2017.06.070 PMID: 28651866

48. Di Foggia G, Beccarello M. Drivers of municipal solid waste management cost based on cost models inherent to sorted and unsorted waste. Waste Management. 2020; 114: 202–214. https://doi.org/10.1016/j.wasman.2020.07.012 PMID: 32679478

49. Dollery B, Wallis J, Allan P. The debate that had to happen but never did: The changing role of Australian local government. Aust J Polit Sci. 2006; 41: 553–567. https://doi.org/10.1080/10361140600959775

50. Tran CDTT, Dollery B. Council type and the determinants of municipal expenditure: A semiparametric analysis of South Australian local government. Australian Journal of Public Administration. 2019; 78: 631–650. https://doi.org/10.1111/1467-8500.12399

51. Allison AL, Lorencatto F, Michie S, Miodownik M. Barriers and Enablers to Food Waste Recycling: A Mixed Methods Study amongst UK Citizens. Int J Environ Res Public Health. 2022; 19: 2729. https://doi.org/10.3390/ijerph19052729 PMID: 35270421

52. Millar L, Bhoyroo R, Molina YP, Watts J, Geagea A, Murphy J, et al. Intersection between health, health literacy and local government: a mixed methods approach to identifying ways to better connect people to place-based primary health care in western Australia. BMC Health Serv Res. 2023; 23: 1–15. https://doi.org/10.1186/S12913-022-08872-9/TABLES/5

53. O'Brien BC, Harris IB, Beckman TJ, Reed DA, Cook DA. Standards for reporting qualitative research: A synthesis of recommendations. Academic Medicine. 2014; 89: 1245–1251. https://doi.org/10.1097/ACM.0000000000000388 PMID: 24979285

54. Qualtrics [Software]. Qualtrics LLC.. Utah, USA: Qualtrics; 2014. https://www.qualtrics.com/

55. Elizabeth Aburn G, Gott M, Hoare K. Experiences of an insider researcher—interviewing your own colleagues. Nurse Res. 2021; 29: 22–28. https://doi.org/10.7748/nr.2021.e1794 PMID: 34137236

56. DeJonckheere M, Vaughn LM. Semistructured interviewing in primary care research: a balance of relationship and rigour. Fam Med Community Health. 2019; 7: 57. https://doi.org/10.1136/fmch-2018-000057 PMID: 32148704

57. Braun V, Clarke V, Boulton E, Davey L, McEvoy C. The online survey as a qualitative research tool. Int J Soc Res Methodol. 2021; 24: 641–654. https://doi.org/10.1080/13645579.2020.1805550

58. Laila A, von Massow M, Bain M, Parizeau K, Haines J. Impact of COVID-19 on food waste behaviour of families: Results from household waste composition audits. Socioecon Plann Sci. 2022; 82: 101188. https://doi.org/10.1016/j.seps.2021.101188 PMID: 34744191

59. Soma T. Space to waste: the influence of income and retail choice on household food consumption and food waste in Indonesia. 2019; 25: 372–392. https://doi.org/10.1080/13563475.2019.1626222

60. Charmaz K. Grounded Theory as an Emergent Methods. In: Nagy Hesse_Biber S, Leavy P, editors. Handbook of Emergent Methods. The Guilford Press; 2008. https://books.google.ca/books?hl=en&lr=&id=vbyxBkkjFeYC&oi=fnd&pg=PA155&dq=charmaz+2008&ots=cP6sWLBleh&sig=UNwoDwX6ZwRtRcmVkSyTRFZMRF4&redir_esc=y#v=onepage&q=charmaz%202008&f=false

61. Saldaña J. Coding techniques for quantitative and mixed data. The Routledge Reviewer's Guide to Mixed Methods Analysis. 2021; 151–160. https://doi.org/10.4324/9780203729434-14

62. Roberts K, Dowell A, Nie J-B. Attempting rigour and replicability in thematic analysis of qualitative research data; a case study of codebook development. BMC Med Res Methodol. 2019; 19. https://doi.org/10.1186/s12874-019-0707-y PMID: 30922220

63. García-Juan B, Escrig-Tena AB, Roca-Puig V. Empowerment in the Public Sector: Testing the Influence of Goal Orientation. Public Pers Manage. 2019; 48: 443–470. https://doi.org/10.1177/0091026018819020

64. Bandura A. Human Agency in Social Cognitive Theory. American Psychologist. 1989; 44: 1175–1184. https://doi.org/10.1037/0003-066x.44.9.1175 PMID: 2782727

65. Singh M, Sarkar A. Role of psychological empowerment in the relationship between structural empowerment and innovative behavior. Management Research Review. 2019; 42: 521–538. https://doi.org/10.1108/MRR-04-2018-0158

66. Bird S, Amarakoon U, Liang X, Pearson D. The vital role of law in fighting Australia's food waste. Alternative Law Journal. 2022; 47: 1–6. https://doi.org/10.1177/1037969X221098483

67. Babbitt CW, Neff RA, Roe BE, Siddiqui S, Chavis C, Trabold TA. Transforming wasted food will require systemic and sustainable infrastructure innovations. Curr Opin Environ Sustain. 2022; 54: 101151. https://doi.org/10.1016/j.cosust.2022.101151

68. NSW Auditor-General. Waste levy and grants for waste infrastructure. Sydney; 2020.

69. Herrfahrdt-Pähle E, Schlüter M, Olsson P, Folke C, Gelcich S, Pahl-Wostl C. Sustainability transformations: socio-political shocks as opportunities for governance transitions. Global Environmental Change. 2020; 63: 102097. https://doi.org/10.1016/j.gloenvcha.2020.102097 PMID: 32801482

70. Galego D, Moulaert F, Brans M, Santinha G. Social innovation & governance: a scoping review. https://doi.org/101080/1351161020211879630. 2021; 35: 265–290. https://doi.org/10.1080/13511610.2021.1879630

71. David Tàbara J, Frantzeskaki N, Hölscher K, Pedde S, Kok K, Lamperti F, et al. Positive tipping points in a rapidly warming world. Curr Opin Environ Sustain. 2018; 31: 120–129. https://doi.org/10.1016/J.COSUST.2018.01.012

72. Moura LN, Camponogara S, Dos Santos JLG, Gasparino RC, Da Silva RM, Freitas EDO. Structural empowerment of nurses in the hospital setting. Rev Lat Am Enfermagem. 2020; 28: e3373. https://doi.org/10.1590/1518-8345.3915.3373 PMID: 33174992

73. Tobin S, Zaman A. Regional Cooperation in Waste Management: Examining Australia's Experience with Inter-municipal Cooperative Partnerships. Sustainability 2022, Vol 14, Page 1578. 2022; 14: 1578. https://doi.org/10.3390/SU14031578

74. Du L, Zuo J, Chang R, Zillante G, Li L, Carbone A. Effectiveness of solid waste management policies in Australia: An Exploratory Study. Environ Impact Assess Rev. 2023; 98: 106966. https://doi.org/10.1016/J.EIAR.2022.106966

75. Webster A, Gardner J. Aligning technology and institutional readiness: The adoption of innovation. Technol Anal Strateg Manag. 2019; 31: 1229–1241. https://doi.org/10.1080/09537325.2019.1601694

76. Ali O, Shrestha A, Ghasemaghaei M, Beydoun G. Assessment of Complexity in Cloud Computing Adoption: A Case Study of Local Governments in Australia. Information Systems Frontiers 2021 24:2. 2021; 24: 595–617. https://doi.org/10.1007/S10796-021-10108-W

77. Coffey B, Bush J, Mumaw L, de Kleyn L, Furlong C, Cretney R. Towards good governance of urban greening: insights from four initiatives in Melbourne, Australia. Australian Geographer. 2020; 51: 189–204. https://doi.org/10.1080/00049182.2019.1708552

78. Busetti S, Pace N. Is this the era of food waste policy? Food Loss and Waste Policy: From Theory to Practice. 2022; 1–10. https://doi.org/10.4324/9781003226932-1/ERA-FOOD-WASTE-POLICY-SIMONE-BUSETTI-NOEMI-PACE

79. Treutwein R, Cleaner NL. Setting the agenda for food waste prevention—A perspective on local government policymaking. J Clean Prod. 2021; 286. https://doi.org/10.1016/j.jclepro.2020.125337

80. NSW EPA. Scrap Together—FOGO education campaign. In: epa.nsw.gov.au [Internet]. 2021 [cited 27 Jan 2022]. https://www.epa.nsw.gov.au/your-environment/recycling-and-reuse/business-government-recycling/food-organics-and-garden-organics/scrap-together

81. Sustainability Victoria. The Path to Half. Victoria; 2020.

82. Kalogiannidis S, Kontsas S, Konteos G, Chatzitheodoridis F. A small City Rebranding: Another View of the Territorial Marketing. WSEAS TRANSACTIONS ON ENVIRONMENT AND DEVELOPMENT. 2022; 18: 686–701. https://doi.org/10.37394/232015.2022.18.65

83. Kansal M, Ananda J, Mitsis A, Karunasena GG, Pearson D. Food Waste in Households: Children as Quiet Powerhouses. Food Qual Prefer. 2022; 98. https://doi.org/10.1016/j.foodqual.2021.104524

84. Ananda J, Karunasena GG, Kansal M, Mitsis A, Pearson D. Quantifying the effects of food management routines on household food waste. J Clean Prod. 2023; 136230. https://doi.org/10.1016/J.JCLEPRO.2023.136230

85. Landells E, Naweed A, Pearson DH, Karunasena GG, Oakden S. Out of Sight, Out of Mind: Using Post-Kerbside Organics Treatment Systems to Engage Australian Communities with Pro-Environmental Household Food Waste Behaviours. Sustainability. 2022 Jul 15; 14(14):8699. https://doi.org/10.3390/su14148699

86. Landells E, Naweed A, Karunasena GG, Pearson DH, Oakden S. Food waste tectonics: Points of friction between policy push and practice pull in council-led household-food-waste interventions in Australia. Journal of Environmental Management. 2024 Apr 1; 357:120717. https://doi.org/10.1016/j.jenvman.2024.120717 PMID: 38579466

87. Policastro G, Cesaro A. Composting of Organic Solid Waste of Municipal Origin: The Role of Research in Enhancing Its Sustainability. International Journal of Environmental Research and Public Health 2023, Vol 20, Page 312. 2022; 20: 312. https://doi.org/10.3390/ijerph20010312 PMID: 36612633
